# Antibody neutralization of cell-surface gC1qR/HABP1/SF2-p32 prevents lamellipodia formation and tumorigenesis

**DOI:** 10.18632/oncotarget.10267

**Published:** 2016-06-24

**Authors:** Beom-Chan Kim, Hyun-Jung Hwang, Hyoung-Tae An, Hyun Lee, Jun-Sub Park, Jin Hong, Jesang Ko, Chungho Kim, Jae-Seon Lee, Young-Gyu Ko

**Affiliations:** ^1^ Tunneling Nanotube Research Center, Korea University, Seoul, 02841, Korea; ^2^ Division of Life Sciences, Korea University, Seoul, 02841, Korea; ^3^ Department of Molecular Medicine, College of Medicine, Inha University, Incheon, 22212, Korea

**Keywords:** gC1qR, lamellipodia, cell migration, antibody, cancer

## Abstract

We previously demonstrated that cell-surface gC1qR is a key regulator of lamellipodia formation and cancer metastasis. Here, we screened a monoclonal mouse antibody against gC1qR to prevent cell migration by neutralizing cell-surface gC1qR. The anti-gC1qR antibody prevented growth factor-stimulated lamellipodia formation, cell migration and focal adhesion kinase activation by inactivating receptor tyrosine kinases (RTKs) in various cancer cells such as A549, MDA-MB-231, MCF7 and HeLa cells. The antibody neutralization of cell-surface gC1qR also inhibited angiogenesis because the anti-gC1qR antibody prevented growth factor-stimulated RTK activation, lamellipodia formation, cell migration and tube formation in HUVEC. In addition, we found that A549 tumorigenesis was reduced in a xenograft mouse model by following the administration of the anti-gC1qR antibody. With these data, we can conclude that the antibody neutralization of cell-surface gC1qR could be a good therapeutic strategy for cancer treatment.

## INTRODUCTION

The receptor for the globular head of complement subunit C1q, gC1qR, is first identified as a binding molecule of C1q and nuclear splicing factor (SF2-p32) [1, 2]. Although gC1qR is mainly localized in the mitochondria, it is also found on the cell-surface in various cell types such as 3T3-L1 adipocytes and A549 cells [3–7]. The cell-surface gC1qR binds to extracellular matrix components such as hyaluronic acid, fibronectin and vitronectin [8–10]. For example, the cell-surface gC1qR associates with hyaluronic acid, which is why it is also called hyaluronic acid-binding protein-1 (HABP1). In addition, the molecular association of gC1qR with C1q leads to classical complement activation [11].

Previous studies have shown that the cell-surface gC1qR could be a diagnostic marker and a new therapeutic target for cancer. The expression level of gC1qR is highly induced in various human malignant tumors, such as lung, breast, ovary, endometrial and colon tumors, compared to their normal tissues [5, 12–14]. The gC1qR expression level is inversely correlated with overall and tumor progression-free survival rate and is in proportion to the cisplatin-resistance in ovarian cancer patients [12]. Notably, the cell-surface gC1qR levels increase in breast cancer cells after tumor xenograft and gradually increased with cancer progression in an MCF10A cancer progression model [5].

Cell migration is a critical process for embryonic development, organogenesis and homeostasis [15]. Uncontrolled regulation of cell migration induces tumor formation and metastasis [16, 17]. Tumor cell migration is mainly stimulated by various growth factors, such as insulin-like growth factor-1 (IGF-1), epithermal growth factor (EGF), hepatocyte growth factor (HGF), and basic fibroblast growth factor (bFGF) [18–21]. For cell migration, mammalian cells initially form lamellipodia that protrude into the direction of movement [22, 23]. The lamellipodia formation is induced by actin nucleation and extension and modulated by the actin-related protein 2/3 (Arp2/3) complex, suppressor of cAMP receptor/Wiskott–Aldrich syndrome protein (WASP) family verprolin-homologous protein (Scar/WAVE), chemokine receptor, CD44, ezrin/radixin/moesin and Rho family GTPases [22–26].

The cell-surface gC1qR is also found in the lamellipodia after cellular exposure to insulin, IGF-1, EGF or fetal bovine serum (FBS) [3]. Lamellipodia formation, cell migration and receptor tyrosine kinase (RTKs including insulin receptor, IGFR and EGFR) signaling is prevented by gC1qR knockdown, indicating that gC1qR is an essential component of lamellipodia formation [3]. Because gC1qR knockdown decreases both mitochondrial and cell-surface gC1qR, it is difficult to conclude that only cell-surface gC1qR is necessary for lamellipodia formation. To overcome this weakness and elucidate the functions of the cell-surface gC1qR, we treated various cancer cells with a monoclonal mouse anti-gC1qR antibody, which was first screened by monitoring cell migration inhibition. In this study, we examined the effect of the antibody neutralization of cell-surface gC1qR in growth factor-stimulated cell migration, lamellipodia formation, proliferation, RTK activation, angiogenesis and *in vivo* tumorigenesis.

## RESULTS

### Antibody neutralization of cell-surface gC1qR prevents cell migration

**Figure 1:**
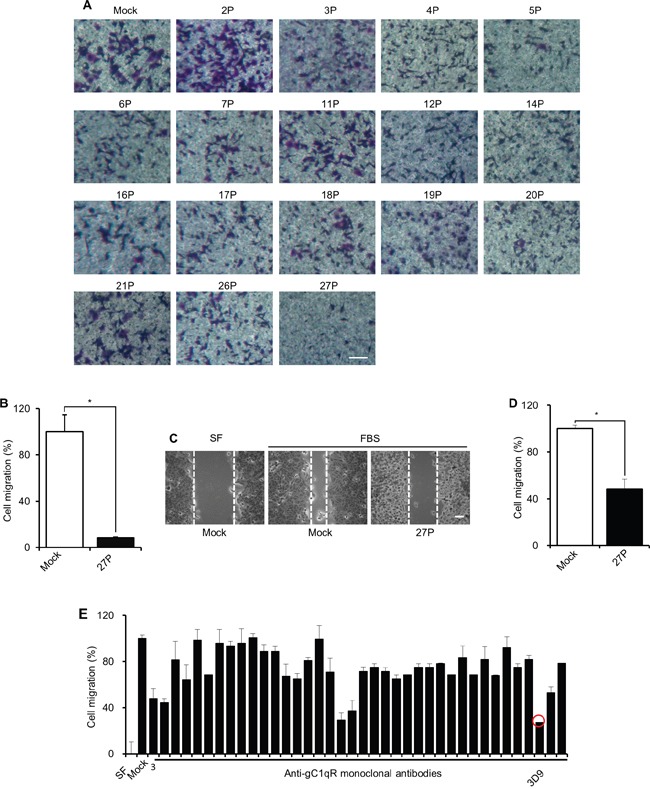
Preparation of a gC1qR-neutralizing antibody **A** and **B.** Different anti-gC1qR antibodies were purified from the cell culture fluid obtained from different hybridoma parental cells. A549 cells were serum-starved for 18 h, pretreated with mock IgG and anti-gC1qR antibodies for 4 h and trypsinized. The A549 cells (4 × 10^4^ cells) were loaded into the upper chamber in trans-well plate in the presence of mock IgG or anti-gC1qR antibodies (10 μg/mL) and stimulated for 18 h by 10% FBS. The trans-well membrane was stained by crystal violet (A). Cell migration was statistically determined in the presence of P27 anti-gC1qR antibody (n=3) (B). **C** and **D.** Cell migration of A549 was determined by wound healing assays. A549 cells were serum-starved for 18 h and pretreated with 10 μg/mL mock IgG or P27 anti-gC1qR antibody for 4 h. The cells were scraped and stimulated by 10% FBS for 30 h. Cells migrating into a wounded area were observed after staining with crystal violet (C). Cell migration was statistically determined (n=3) (D). **E.** P27 parental cells were further sub-cloned by semi-solid cloning. Anti-gC1qR monoclonal mouse antibodies were prepared from the cell culture media from each sub-clone. Cell migration of A549 cells was statistically determined by wound healing assay in the presence of mock IgG or anti-gC1qR antibody (10 μg/mL) (n=3). Scale bar = 100 μm. Graphs represent mean ± standard error of the mean (s.e.m.) *p < 0.01, student *t* test.

Because cell-surface gC1qR is known to increase during cancer progression and regulates lamellipodia formation and cell migration [3, 5, 12], antibody neutralization of cell-surface gC1qR might be an effective strategy for treating cancer. To identify cell-surface gC1qR-neutralizing antibodies, we screened anti-gC1qR mouse antibodies using trans-well migration assays. Fetal bovine serum (FBS)-induced A549 cell migration was monitored in trans-wells after incubation with anti-gC1qR antibody obtained from different parental hybridoma cells. As shown in Figure 1A and 1B, anti-gC1qR antibody from parental hybridoma cell line number 27 (P27) was identified as the most effective in cell migration inhibition. The P27 anti-gC1qR antibody also prevented FBS-induced cell migration in wound-healing assays (Figure 1C and 1D). The relative migration was reduced up to ~90% in trans-well migration assays and ~50% in wound-healing assays by P27 anti-gC1qR antibody compared to mock IgG (Figure 1B and 1D). Next, the P27 cells were further cloned a second time using semi-solid cloning to obtain optimal monoclonal mouse anti-gC1qR antibodies (mAb) for cell migration inhibition. FBS-induced A549 cell migration was monitored in wound healing assay after pre-incubating the cells with mock IgG or monoclonal mouse anti-gC1qR antibodies obtained from each clone. Because mAb 3D9 was the most effective antibody at preventing FBS-stimulated cell migration of A549 cells (Figure 1E), we used the mAb 3D9 to neutralize cell-surface gC1qR in further experiments.

**Figure 2:**
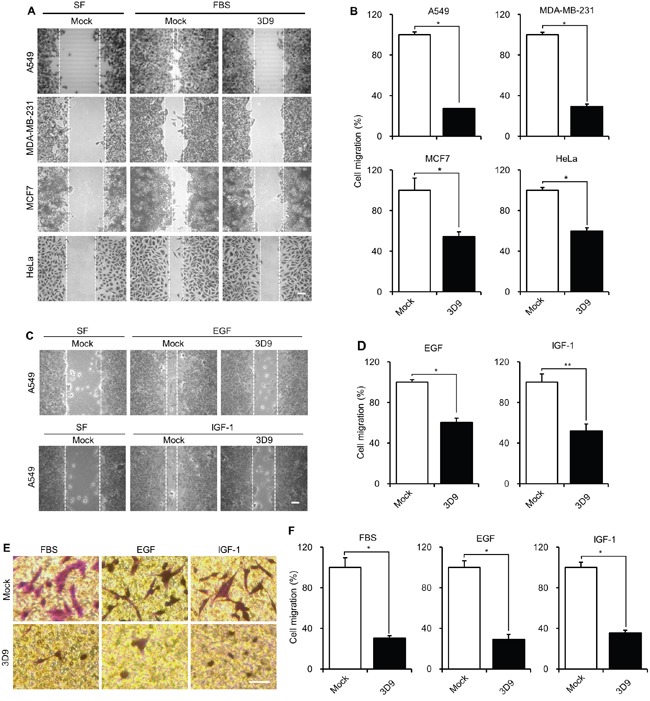
Antibody neutralization of gC1qR prevents cell migration **A** and **B.** A549, MDA-MB-231, MCF7 and HeLa cells were serum-starved for 18 h and pretreated with 10 μg/mL of mock IgG or anti-gC1qR antibody (mAb 3D9) for 4 h. FBS-induced cell migration was determined by wound-healing assays after stimulating cells with FBS (10%) for 30 h with A549, MCF7 and MDA-MB-231 cells and for 12 h with HeLa cells (A). Cell migration was statistically determined (n=3) (B). **C** and **D.** EGF- and IGF-1-induced cell migration was determined by wound-healing assay. A549 cells were serum-starved for 18 h, pretreated with 10 μg/mL of mock IgG or mAb 3D9 for 4 h and stimulated with EGF (50 ng/mL) or IGF-1 (100 ng/mL) for 30 h (C). Cell migration was statistically determined (n=3) (D). **E** and **F.** FBS-, EGF- and IGF-1-induced cell migration of A549 cells was determined by trans-well assay in the presence of mock IgG or mAb 3D9 (E). Cell migration was statistically determined (n=3) (F). Scale bar = 100 μm. Graphs represent mean ± s.e.m. *p < 0.01, student *t* test.

Next, we tested whether mAb 3D9 inhibits FBS-induced cell migration in various cancer cell lines, such as human breast carcinoma MDA-MB-231, human breast carcinoma MCF7, human cervix carcinoma HeLa and human lung carcinoma A549 cells, which expressed gC1qR in the plasma membrane and mitochondria (Supplementary Figure 1A). In the wound healing assay, mAb 3D9 inhibited FBS-induced cell migration of HeLa, MCF7, A549 and MDA-MB-231 cells (Figure 2A and 2B). Notably, the FBS-induced cell migration was dramatically reduced by mAb 3D9 in A549 and MDA-MB-231 cells, which highly expressed gC1qR in the plasma membrane (Supplementary Figure 1A). Thus, A549 and MDA-MB-231 cell lines were selected for further investigating the effect of mAb 3D9 on cell migration inhibition.

We examined EGF- and IGF-1-induced cell migration of A549 and MDA-MB-231 cells in the presence of mAb 3D9. In wound-healing assays of both cells, EGF- and IGF-1-induced cell migration was significantly inhibited by mAb 3D9 (Figure 2C and 2D and Supplementary Figure 1B and 1C). We also confirmed that mAb 3D9 inhibited FBS-, EGF- and IGF-1-induced cell migration in trans-well migration assays of both cells (Figure 2E and 2F and Supplementary Figure 1D and 1E). These results suggest that mAb 3D9 is useful for neutralizing the cell-surface gC1qR in various cancer cells.

### Antibody neutralization of cell-surface gC1qR prevents lamellipodia formation

**Figure 3:**
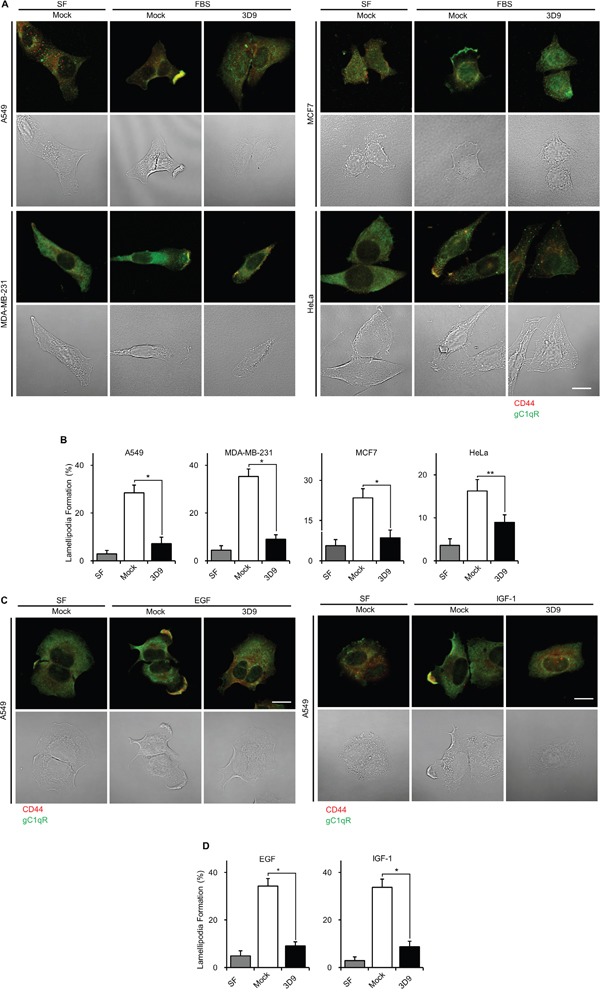
Antibody neutralization of gC1qR prevents lamellipodia formation **A** and **B.** MDA-MB231, A549, MCF7 and HeLa cells were grown to non-confluency, serum-starved for 18 h, pretreated with 10 μg/mL of mock IgG or mAb 3D9 for 4 h and stimulated for 10 min by 10% FBS. Cellular localization of gC1qR and CD44 was determined by non-permeabilized immunofluorescence (A). Lamellipodia-containing cells (%) were statistically determined from 12 different imaging fields (B). **C** and **D.** A549 cells were grown to non-confluency, serum-starved for 18 h, pretreated with 10 μg/mL of mock IgG or mAb 3D9 for 4 h and stimulated for 10 min by EGF (50 ng/mL) and IGF-1 (100 ng/mL) for 10 min. Cellular localization of gC1qR and CD44 was determined by non-permeabilized immunofluorescence (C). Lamellipodia-containing cells (%) were statistically determined from 12 different imaging fields (D). Scale bar = 10 μm. Graphs represent mean ± s.e.m. *p < 0.01 and **p < 0.05, student *t* test.

It is known that cell-surface gC1qR is a key regulator for lamellipodia formation in A549 cells [3]. To assess the involvement of cell-surface gC1qR in lamellipodia formation, we investigated the gC1qR and CD44 localization of lamellipodia in various non-permeabilized cancer cells using mAb 3D9. CD44 was used as a cell-surface marker of lamellipodia. As shown in Figure 3A, cell-surface gC1qR and CD44 were dispersed on the cell-surface of serum-starved and mock IgG-treated A549, MDA-MB-231, MCF7 and HeLa cells. After FBS stimulation in the presence of mock IgG, the cell-surface gC1qR and CD44 appeared in the lamellipodia in all tested cell lines. In the presence of mAb 3D9, the gC1qR and CD44-containing lamellipodia disappeared even with FBS stimulation, indicating that mAb 3D9 prevents FBS-stimulated lamellipodia formation in various cell lines (Figure 3A). The mAb 3D9 had the strongest inhibitory effect on lamellipodia formation in A549 and MDA-MB-231 cells (Figure 3B). In addition, EGF- and IGF-1-stimulated lamellipodia formation in A549 cells was prevented by mAb 3D9 (Figure 3C and 3D). These data suggest that mAb 3D9 prevents FBS-, EGF- or IGF-1-stimulated lamellipodia formation by neutralizing cell-surface gC1qR.

### Antibody neutralization of cell-surface gC1qR prevents the activation of RTKs

**Figure 4:**
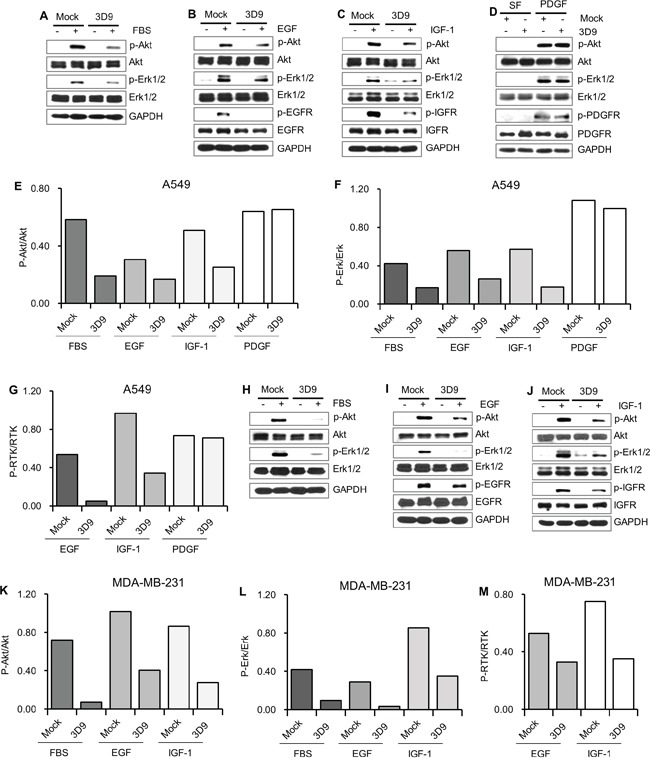
Antibody neutralization of gC1qR prevents receptor tyrosine kinases (RTKs) signaling **A-D.** A549 cells were serum-starved for 18 h and pretreated with 10 μg/mL of mock IgG or mAb 3D9 for 4 h and stimulated for 10 min by FBS (10%) (A), EGF (50 ng/mL) (B), IGF-1 (100 ng/mL) (C) and PDGF (20 ng/mL) (D) for 10 min. **H-J.** MDA-MB-231 cells were serum-starved for 18 h and pretreated with 10 μg/mL of mock IgG or mAb 3D9 for 4 h and stimulated for 10 min by FBS (10%) (H), EGF (50 ng/mL) (I) and IGF-1 (100 ng/mL) (J). Phosphorylated and total forms of Akt, Erk1/2, EGFR, IGFR and PDGFR were analyzed by immunoblotting using GAPDH as a loading control. The ratio of p-Akt/Akt **E and K.**, p-Erk/Erk **F and L.**, p-EGFR/EGFR **G and M.**, p-IGFR/IGFR (G and M) and p-PDGFR/PDGFR (G) was calculated from the band intensities for p-Akt, Akt, p-Erk1/2, Erk1/2, p-EGFR, EGFR, p-IGFR, IGFR, p-PDGFR and PDGFR in the immunoblottings (A-D and H-J).

Next, we tested whether mAb 3D9 affects the FBS, EGF and IGF-1 signal transduction pathways. In the presence of mock IgG or mAb 3D9, the phosphorylation of the RTKs, Akt and Erk was monitored in FBS-, EGF- and IGF-1-stimulated A549 and MDA-MB-231 cells. In contrast to mock IgG-pretreated cells, IGFR, EGFR, Akt and Erk phosphorylation was significantly reduced in mAb 3D9-pretreated A549 (Figure 4A-4C and Figure 4E-4G) and MDA-MB-231 cells (Figure 4H-4J and Figure 4K-4M). We also monitored PDGF signaling in mock IgG- or mAb 3D9-treated A549 cells. As shown in Figure 4D and Figure 4E-4G, there was no difference in the phosphorylation of PDGFR, Akt and Erk between mock IgG- and mAb 3D9-treated A549 cells. These data suggest that cell-surface gC1qR neutralization with mAb 3D9 prevents the activation of IGFR and EGFR but not PDGFR.

### Antibody neutralization of cell-surface gC1qR prevents the activation of focal adhesion kinase

**Figure 5:**
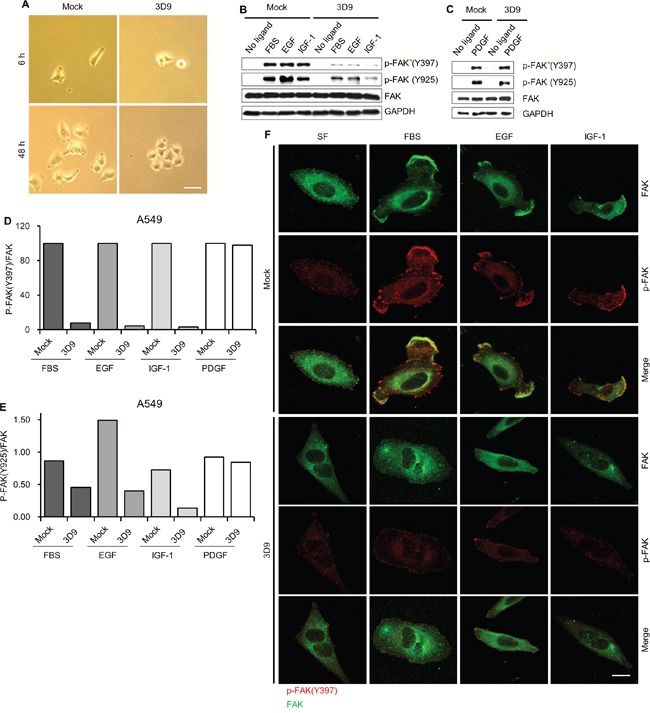
Antibody neutralization of gC1qR prevents the activation of focal adhesion kinase (FAK) **A.** A549 cells were grown after plating in the presence of 10 μg/mL of mock IgG or mAb 3D9. Cellular morphology was observed with a light microscopy 6 h and 48 h after cell plating. Scale bar = 50 μm. **B** and **C.** A549 was serum-starved for 18 h and pretreated with 10 μg/mL of mock IgG or mAb 3D9 for 4 h and stimulated for 10 min by FBS (10%), EGF (50 ng/mL), IGF-1 (100 ng/mL) and PDGF (20ng/mL) for 10 min. Phosphorylated (at Y397 or Y925) and total forms of FAK were analyzed by immunoblotting using GAPDH as a loading control (B and C). The ratio of p-FAK/FAK **D and E.** was calculated from the band intensities for p-FAK (Y397), p-FAK (Y925) and FAK in the immunoblottings (B and C). Cellular localization of p-FAK (Y397) and FAK was determined by immunofluorescence **F.** Scale bar = 10 μm.

Because cell-surface gC1qR binds to various extracellular matrix components [3, 8-10, 27], antibody neutralization of cell-surface gC1qR might affect cell adhesion and spreading. We observed the cellular morphology of A549 after cell plating in the presence of mock IgG or mAb 3D9. Cell spreading was delayed in the presence of mAb 3D9 at 6 h after cell plating (Figure 5A, two upper panels). Interestingly, mock IgG-treated cells showed a dispersed growth pattern whereas mAb 3D9-treated cells had an aggregated growth pattern at 48 h after cell plating (Figure 5A, two lower panels). In the presence of mAb 3D9, the activation of focal adhesion kinase (FAK) was determined by immunoblotting and immunofluorescence. As shown in Figure 5B-5F, mAb 3D9 dramatically reduced FAK phosphorylation at Y397 and Y925 in FBS-, EGF-, and IGF-1-stimulated A549 cells but not in PDGF-stimulated cells. On the basis of these data, we conclude that the antibody neutralization of cell-surface gC1qR prevents FAK activation stimulated with FBS-, EGF-, and IGF-1 but not PDGF.

### Antibody neutralization of cell-surface gC1qR prevents angiogenesis in HUVEC

**Figure 6:**
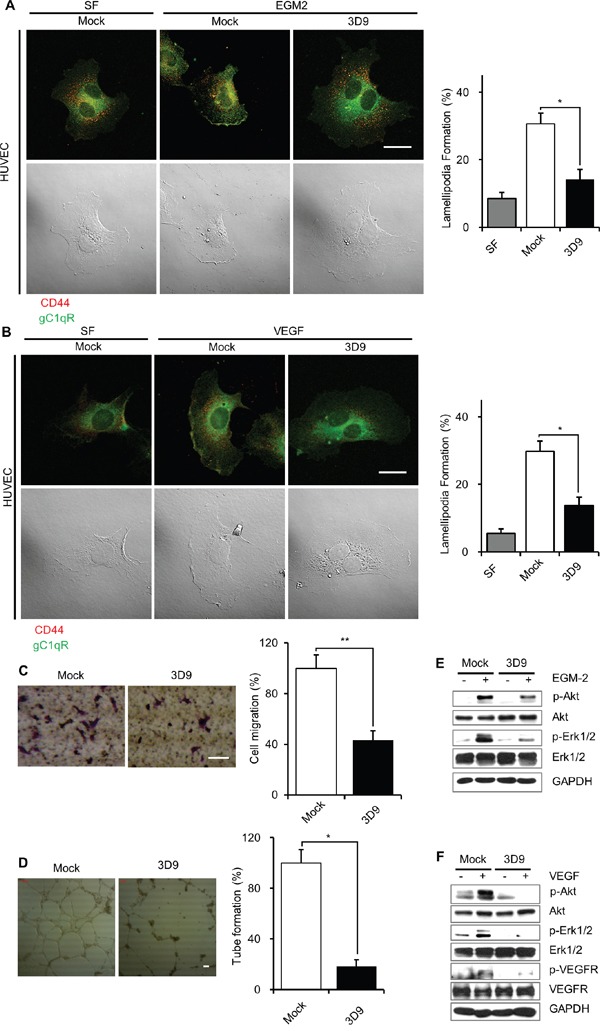
Antibody neutralization of gC1qR prevents lamellipodia formation, cell migration and VEGF signaling in HUVEC **A** and **B.** Human umbilical vein endothelial cells (HUVEC) were grown to non-confluency, serum-starved with 0.2% FBS-containing endothelial basal media (EBM-2) for 18 h, pretreated with 10 μg/mL of mock IgG or mAb 3D9 for 4 h and stimulated with endothelial growth media (EGM-2) (A) or vascular endothelial growth factor (VEGF, 50 ng/mL) (B) for 10 min. Cellular localization of gC1qR and CD44 was determined by non-permeabilized immunofluorescence (right panels). Lamellipodia-containing cells (%) were statistically determined from 12 different imaging fields (left panels). Bar = 20 μm. **C.** EGM-2-induced cell migration of HUVEC was determined by trans-well assay in the presence of 10 μg/mL of mock IgG or mAb 3D9. The trans-well membrane was stained by crystal violet (left panel). Cell migration was statistically determined from three independent experiments (right panel). Bar = 50 μm. **D.** Serum-starved HUVEC were serum-starved in 0.2% FBS-containing EBM-2 for 18 h and trypsinized. The cells were loaded into a Matrigel-coated cell plate and tube formation was induced with EGM-2 for 18 h in the presence of 200 μg/mL mock IgG or mAb 3D9. Scale bar = 100 μm. **E and F.** Serum-starved HUVEC were pretreated with 10 μg/mL of mock IgG or mAb 3D9 for 4 h and stimulated by EGM-2 (E) or VEGF (50 ng/mL) (F) for 10 min. Phosphorylated and total forms of Akt, Erk1/2 and VEGFR were analyzed by immunoblotting using GAPDH as a loading control. *p < 0.01 and **p < 0.05, student *t* test.

Because gC1qR is expressed on the cell-surface in endothelial cells [5, 7, 28], it is tempting to speculate that the antibody neutralization of cell-surface gC1qR might inhibit angiogenesis in endothelial cells. To address the issue, we investigated the effect of mAb 3D9 on lamellipodia formation, cell migration, tube formation and vascular endothelial growth factor (VEGF) signaling in human umbilical vein endothelial cells (HUVEC). The lamellipodial localization of cell-surface gC1qR and CD44 were observed in the HUVEC stimulated by EGM-2 containing endothelial growth factors or VEGF in the presence of mock IgG but not in the presence of mAb 3D9 (Figure 6A and 6B). The neutralization of cell-surface gC1qR with mAb 3D9 significantly reduced EGM-2-stimulated HUVEC migration and tube formation as determined by trans-well migration and tube formation assays, respectively (Figure 6C and 6D). The mAb 3D9 also decreased EGM-2- or VEGF-stimulated phosphorylation of VEGFR, Akt and Erk (Figure 6E and 6F). Based on the data in HUVEC, we conclude that mAb 3D9 could be used as an anti-angiogenic drug for cancer treatment.

### Antibody neutralization of cell-surface gC1qR prevents *in vivo* tumorigenesis

**Figure 7:**
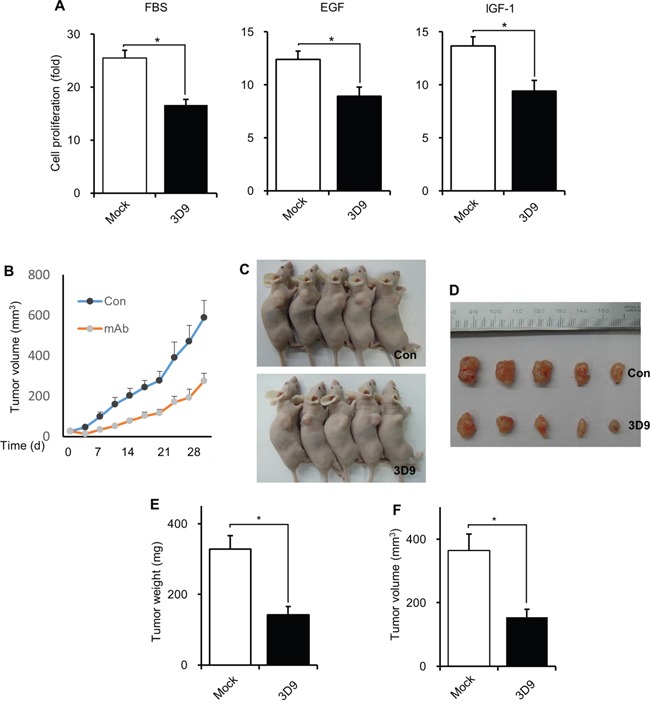
Antibody neutralization of gC1qR prevents *in vivo* tumor growth **A.** A549 cells (2×10^4^) were seeded into 6-well plate in the presence 10 μg/mL of mock IgG or mAb 3D9 and further grown for 4 days after adding FBS (10%), EGF (50 ng/mL) or IGF-1 (100 ng/mL). Cell number was counted from three independent experiments using a hemocytometer. **B-D.** A549 cells (3 × 10^6^) were subcutaneously injected into BALB/c athymic mice (n=5). mAb 3D9 (10 mg/kg) or PBS (control) was intraperitoneally injected twice a week in the tumor-bearing mice. Tumor volume was measured twice a week until day 32 (B) and photographs were taken on day 32 (C and D). *p < 0.01. **E** and **F.** Tumor weight (E) and volume (F) were statistically analyzed after tumors were isolated from mice at day 32. *p < 0.01, student *t* test.

We investigated whether antibody neutralization of cell-surface gC1qR inhibits growth factors-induced cellular proliferation. As shown in Figure 7A, mAb 3D9 decreased FBS-, EGF-, or IGF-1-stimulated cellular proliferation of A549 cells. To explore the possibility that mAb 3D9 could be used as an anti-cancer drug, we next examined whether mAb 3D9 could affect *in vivo* tumor growth after A549 cells were subcutaneously injected into BALB/c athymic mice. Tumor size was measured for 32 days after A549 tumor-bearing mice were treated twice a week with PBS or mAb 3D9. The administration of mAb 3D9 delayed tumor growth (Figure 7B). mAb 3D9-treated mice showed reduced tumor weights and volumes after 32 days, compared to PBS-treated mice (Figure 7C-7F). These results suggest that the mAb 3D9 could be used as an anti-cancer drug.

## DISCUSSION

Although gC1qR is first identified as a receptor of the globular head for complement subunit C1q and as a splicing factor-associated protein, the gC1qR is mainly localized in the mitochondria and maintains mitochondrial structure and function [1, 2, 29]. The systemic disruption of gC1qR reduces the expression level of mitochondrial oxidative phosphorylation (OXPHOS) subunits, leading to mitochondrial dysfunction with reduced mitochondrial respiration [29, 30]. These findings suggest that gC1qR is a major mitochondrial protein, which is necessary for mitochondrial integrity and function. In spite of its mitochondrial localization, gC1qR has been identified on the cell-surface of various mammalian cells [2–7]. Because gC1qR interacts with plasma proteins, extracellular matrix molecules and different pathogens [2, 6, 8-10, 31, 32], gC1qR is suggested to localize in the cell-surface or extracellular space. Indeed, gC1qR is found on the cell-surface of non-permeabilized 3T3-L1 adipocytes and various cancer cells, including A549, MDA-MB435 and MDA-MB231 cells as determined by immunofluorescence or FACS analysis [3–5]. Fluorescently labeled Lyp-1 interacts with gC1qR on the cell-surface after its intravenous administration in mice [33]. Interestingly, recombinant gC1qR is also found on the cell-surface, leading to cell migration via interaction with αvβ3 integrin in B16F10 cells [34]. In addition, cell survival and tumorigenicity are enhanced via the molecular interaction between gC1qR and hyaluronic acid (HA) in gC1qR-overexpressing HepG2 cells [35]. These data indicate that gC1qR is a genuine cell-surface protein.

The cell-surface gC1qR plays an important role in lamellipodia formation because it is found in growth factor-induced lamellipodia and gC1qR knockdown prevents lamellipodia formation, cell migration, cell attachment and RTK activation in A549 cells [3]. Our present data also show that antibody neutralization of cell-surface gC1qR prevented ligand-induced lamellipodia formation, cell migration, cell attachment and RTK signaling in various cell lines such as A549, MDA-MB-231, MCF7, HeLa and HUVEC, suggesting that antibody neutralization of cell-surface gC1qR can be a useful treatment strategy for various cancers that overexpress gC1qR and RTKs in the plasma membrane. Indeed, mAb 3D9 reduced tumor growth in A549 tumor-bearing nude mice.

It is tempting to speculate that integrin might be a mediator for gC1qR-induced RTK activation, lamellipodia formation and cell migration because of an integrin-RTK crosstalk [36, 37] and the molecular interaction of integrin α_V_β_3_ with exogenous gC1qR [34]. The administration of recombinant gC1qR induces cell migration via integrin- and NFκB-dependent matrix metalloprotease-2 (MMP-2) activation in B16F10 mouse melanoma cells [34]. Because the recombinant gC1qR-induced cell migration was prevented in the presence of anti- α_V_β_3_ integrin antibody or α_V_β_3_-binding peptide GRGDSP, α_V_β_3_ integrin might be required for the exogenous gC1qR-induced cell migration [34]. However, in A549 cells with low expression level of α_V_β_3_ integrin [38], antibody neutralization of surface gC1qR prevented growth factors-induced lamellipodia formation and cell migration. We also failed to demonstrate the molecular interaction between endogenous gC1qR and α_V_β_3_ integrin in different mammalian cells stimulated IGF or EGF. Thus, it seems that integrin α_V_β_3_ integrin may not be involved in gC1qR-mediated RTK activation, lamellipodia formation, cell migration and tumorigenesis, at least in our model system, A549 cells. However, we do not rule out the possibility of the involvement of integrin α_V_β_3_ in those cellular events in other system.

Because cell-surface CD44 did not appeared in the lamellipodia of growth factor-stimulated cells after antibody neutralization of gC1qR, CD44 might be involved in gC1qR-mediated lamellipodia formation. Interestingly, both gC1qR and CD44 are critical components for lamellipodia formation, because they interact with hyaluronic acid and regulate various RTKs [3, 39, 40]. In addition, there has been a suggestion for a molecular interaction between gC1qR and CD44 [6, 41], speculating that molecular association of gC1qR with CD44 is necessary for lamellipodia formation and RTK activation. However, CD44 knockdown increases PDGF signaling whereas antibody neutralization of cell-surface gC1qR did not change PDGF signaling [42]. This discrepancy of CD44 and gC1qR in PDGF signaling challenges the relationship between CD44 and gC1qR in the regulation of RTK activation and molecular mechanisms of gC1qR in RTK regulation should be carefully investigated without molecular association of gC1qR with CD44.

## MATERIALS AND METHODS

### Cell lines and cell culture

A549, HeLa, MDA-MB-231 and MCF7 cells were obtained from the American Type Culture Collection (ATCC, VA, USA) and HUVEC from Lonza Inc. (Basel, Switzerland). MCF7, HeLa and hybridoma cells were cultured in DMEM (Hyclone, UT, USA) with 10% fetal bovine serum (FBS). A549 and MDA-MB-231 cells were maintained in RPMI (Hyclone, UT, USA) 1640 media containing 10% FBS and HUVEC in EGM-2 (Lonza, Basel, Switzerland).

### Antibodies and reagents

Monoclonal mouse anti-gC1qR antibodies (3D9 and others) were generated using full-length recombinant gC1qR as an antigen. Anti-phospho-Akt, FAK, phospho-FAK, phospho-IGFR, VEGF, phospho-VEGF, phospho-PDGF, PDGF and PDH antibodies were purchased from Cell Signaling (CA, USA) and anti-GAPDH, EGFR, Erk, CD44, flotilin-1, phospho-EGFR and phospho-Erk antibodies from Santa Cruz (CA, USA). Alexa 488- and 555-conjugated secondary antibodies were purchased from Invitrogen (CA, USA). Human recombinant IGF-1, PDGF and VEGF were purchased from R&D Systems (MN, USA) and EGF from Sigma (MO, USA).

### Preparation of monoclonal mouse anti-gC1qR antibody

Monoclonal mouse anti-gC1qR antibodies were raised using recombinant full-length human gC1qR as an antigen. A single-cell hybridoma was isolated with the ClonaCell-HY Hybridoma Cloning Kit (Stemcell technologies, BC, Canada) according to the manufacturer's instruction and screened by ELISA with recombinant gC1qR. Monoclonal antibodies were purified from each hybridoma clone using protein-G affinity chromatography (GE healthcare). Specifically, 3D9 mAb was purified from the media of hybridoma cells (3D9), which were cultured in 8 mM L-glutamine (Sigma, MO, USA)-containing CD hybridoma medium (NY, USA) in Integra CL 1000 flasks (Integra Biosciences, NH, USA) following the manufacturer's instructions.

### Immunoblotting and immunofluorescence

Cells were washed three times with cold PBS and lysed with RIPA buffer (25 mM Tris-HCL. pH 7.6, 150 mM NaCl, 1% NP-40, 1% sodium deoxycholate and 0.1% SDS) containing protease inhibitors (GenDEPOT, TX, USA) and phosphatase inhibitors (GenDEPOT, TX, USA). The cell lysates were separated using SDS-PAGE and transferred to a nitrocellulose membrane. After blocking with 5% (w/v) BSA or skim milk for 1 h in Tris-buffered saline (TBS) with 0.05% Tween 20, primary antibodies were incubated for 1 h at room temperature or overnight at 2-8°C. Next, secondary antibodies were incubated for 1 h and the target proteins were detected with x-ray film in a darkroom.

For immunofluorescence, target cells were fixed with 3.7% para-formaldehyde for 15 min, permeabilized in 0.1% Triton X-100 for 5 min and blocked with PBS containing 5% BSA for 1 h. Primary and secondary antibodies were incubated for 1 h for immunofluorescence. The fluorescence-labeled cells were analyzed with a Zeiss LSM700 Meta confocal microscope (Carl Zeiss, Germany).

### Cell proliferation

For cell proliferation assay, 2.0 × 10^4^ cells were dispensed in a 6-well plate and incubated for 12 h. After the serum starvation for 18 h, the cells were further incubated with FBS (10%)-, EGF (50 ng/mL)- or IGF-1 (100 ng/mL)-containing RPMI in the presence of 10 μg/mL of mock IgG or mAb gC1qR, respectively for 4 days.

### Trans-well migration assay

First, 5×10^4^ cells were serum-starved for 18 h, trypsinized for 4 min, pretreated with 10 μg/mL of mock IgG or anti-gC1qR antibodies for 30 min at 4°C and transferred to the upper chamber of a Boyden chamber. The cells were further incubated for 18 h after FBS (10%)-, EGF (50 ng/mL)- or IGF-1 (100 ng/mL)-containing media (600 μL) were added to the lower chamber. The cells on the membrane were fixed with 3.7% formaldehyde for 20 min and stained with 0.1% crystal violet for 20 min. Cell migration number was counted after removing the upper chamber cells with a cotton swab.

### Wound-healing assay

Wound healing assay was performed as previously described [43]. Cells were grown to confluency, serum-starved for 18 h and pretreated with 10 μg/mL of mock IgG or anti-gC1qR antibodies for 4 h. The cells were scraped with a sterile micropipette tip and stimulated with FBS (10%), EGF (50 ng/mL) or IGF-1 (100 ng/mL) for 12 or 30 h in the presence of 10 μg/mL of mock IgG or anti-gC1qR antibody. The cells were fixed with 3.7% formaldehyde for 20 min and stained with 0.1% crystal violet for 20 min. Images for migrated cells were captured with a microscope.

### Subcellular fractionation

The plasma membrane was isolated according to Hubbard *et al.* (1983) [44], with minor modifications. Cells were harvested with cold TES buffer (20 mM Tris HCl, pH 7.4, 1 mM EDTA, 8.7% sucrose) and homogenized with a glass homogenizer. The homogenized samples were centrifuged at 17,500 rpm in a SW41Ti (Beckman) for 30 min at 4°C. The pellets were re-suspended with TES buffer, loaded on a 38% sucrose cushion and centrifuged at 100,000 x g for 30 min at 4°C. The plasma membranes were isolated from a band between 8.7% and 38% sucrose and mitochondria from a band between 38% and 50% sucrose. The isolated samples were re-suspended with TES buffer and centrifuged at 17500 rpm in a SW41Ti rotor for 30 min at 4°C for 2 times. The pellets were lysed with RIPA buffer for immunoblotting.

### *In vitro* tube formation assay

*In vitro* tube formation assay were performed according to Arnaouova *et al*. [45]. HEVEC were serum-starved in 0.2% FBS-containing EBM-2 media (Lonza, Basel, Switzerland) for 18 h. The cells were dispensed into Matrigel-coated culture plates with EGM-2 media in the presence of 200 μg/mL of mock IgG or anti-gC1qR antibody and incubated for 18 h. The cells were fixed with 3.7% formaldehyde and cell images were taken under a microscope.

### Animal study and *in vivo* tumorigenesis study

Five-week-old female BALB/c nude mice were purchased from Orient Bio Inc. (Seongnam, South Korea) and maintained at 22 ± 2°C and 50 ± 10% humidity under a 12 h light: 12 h dark regimen. The Institutional Animal Care and Use Committee of the Korea Institute of Radiological and Medical Science approved the studies, which were performed under the guidelines for the care and use of laboratory animals. A549 cells (3 × 10^6^) were subcutaneously injected into the right foreleg of mice (*n* = 5/group). One hundred microliters of PBS or anti-gC1qR antibody (10 mg/kg) was intraperitoneally injected twice weekly when palpable tumor developed. Tumors were measured twice weekly for 32 days and tumor size was calculated according to the following equation: V = 0.5 × (width^2^ × length).

## SUPPLEMENTARY MATERIALS FIGURES


