# Supplementary material for: Antibody neutralization of cell-surface gC1qR/HABP1/SF2-p32 prevents lamellipodia formation and tumorigenesis
Source: Oncotarget. 2016 Jun 24;7(31):49972–85. doi: 10.18632/oncotarget.10267 (PMC5226562; doi:10.18632/oncotarget.10267)
Supplement: Supplementary file 1 [file oncotarget-07-49972-s001.pdf]

# Antibody neutralization of cell-surface gC1qR/HABP1/SF2-p32 prevents lamellipodia formation and tumorigenesis

## SUPPLEMENTARY FIGURE

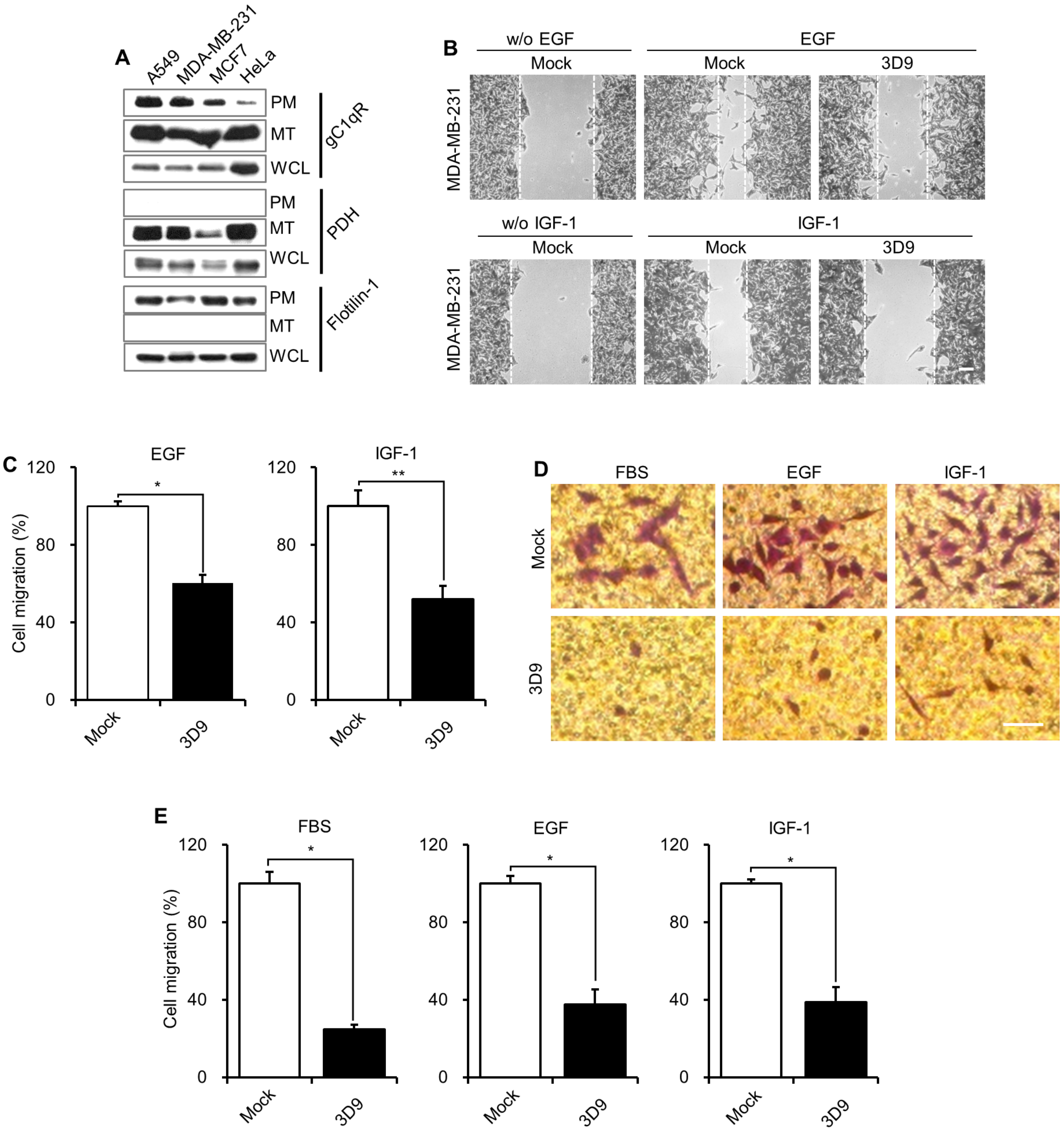


**Supplementary Figure S1: Antibody neutralization of gC1qR prevents cell migration. A.** A549, MDA-MB-231, MCF7 and HeLa cells were scrapped after cold PBS washing, and then homogenized with a glass homogenizer. The plasma membranes and mitochondrias of the cells were isolated using the sucrose gradient method. gC1qR, PDH and flotilin-1 were analyzed by immunoblotting. PM, plasma-membrane; MT, mitochondria; WCL, whole cell lysate. **B** and **C.** MDA-MB-231 cells were serum-starved for 18 h and pretreated with 10 μg/mL of mock IgG or anti-gC1qR antibody (mAb 3D9) for 4 h. EGF- and IGF-1-induced cell migration was determined by wound-healing assays in the presence of 10 μg/mL of mock IgG or mAb 3D9 after stimulating MDA-MB-231 cells with EGF (50 ng/mL) or IGF-1 (100 ng/mL) for 30 h (B). Cell migration was statistically determined (n=3) (C). **D** and **E.** FBS-, EGF- and IGF-1-induced cell migration of MDA-MB-231 was determined by trans-well assays in the presence of mock IgG or mAb 3D9 (D). Cell migration was statistically determined (n=3) (E). Scale bar = 100 μm. *$p < 0.01$, student *t* test.
